# Enhancement of immunomodulative effect of lactic acid bacteria on plasmacytoid dendritic cells with sucrose palmitate

**DOI:** 10.1038/s41598-018-21527-2

**Published:** 2018-02-16

**Authors:** Masaya Kanayama, Yukiko Kato, Toshikazu Tsuji, Yuki Konoeda, Akiko Hashimoto, Osamu Kanauchi, Toshio Fujii, Daisuke Fujiwara

**Affiliations:** 1 Research Laboratories for Health Science & Food Technologies, Kirin Company, Limited, Yokohama, Kanagawa Japan; 2 Central Laboratories for Key Technologies, Kirin Company, Limited, Yokohama, Kanagawa Japan

## Abstract

Plasmacytoid dendritic cells (pDCs) play a key role in the immune response against viruses. In addition, recent research has suggested that pDCs possess direct and indirect tumoricidal activities. We previously found that a lactic acid bacteria strain, *Lactococcus lactis* JCM 5805 (LC-Plasma), stimulated pDCs and prevented viral infection in mouse and human studies. Meanwhile, emulsifiers have recently been highlighted as candidate adjuvants for some viral vaccines and cancer immunotherapies. In this study, we discovered some specific emulsifiers, mainly consisting of sucrose fatty acid esters, that drastically enhance the potency of LC-Plasma to activate pDCs *in vitro*. The emulsifiers promoted the efficient uptake of LC-Plasma by pDCs and the ratio of pDCs that took up LC-Plasma correlated with the activity of pDCs. In addition, an *in vivo* study showed that oral treatment with LC-Plasma mixed with an emulsifier induced a higher expression of genes related to anti-viral immunity in the lung compared to treatment with LC-Plasma alone. Both LC-Plasma and the emulsifiers used in this study have been confirmed to be safe for human use. Therefore, LC-Plasma mixed with an emulsifier might be a useful tool for certain anti-cancer and anti-viral therapies.

## Introduction

Dendritic cells (DCs) are a group of innate immune cells that are broadly divided into two subpopulations, myeloid DCs (mDCs) and plasmacytoid DCs (pDCs). pDCs play a key role in the immune response against viruses by producing large amounts of type I interferons (IFNs)^1,2^ and inducing the expression of numerous interferon-stimulated genes (ISGs) that are responsible for inhibiting viral replication and transmission^3^. pDCs also modify anti-viral immunity by regulating the activities of various immune cells, including natural killer (NK) cells^4^, B cells^5,6^ and T cells^7^. Therefore, it is thought that stimulating the activity of pDCs protects the host from viral infection.

Although some pathogenic bacteria have been shown to stimulate pDCs^8^, beneficial bacteria have been shown to be ineffective in stimulating pDCs^9^. However, we previously screened non-pathogenic lactic acid bacteria (LAB) strains and found that some spherical LAB strains, especially *Lactococcus lactis* subsp*. lactis* JCM 5805, stimulated murine pDCs to produce Type I IFNs in association with mDCs via the Toll-like receptor (TLR) 9/myeloid differentiation primary response gene 88 (MyD88) pathway^10^. *Lactococcus lactis* strain Plasma (LC-Plasma) is a synonym of *Lactococcus lactis* subsp. *lactis* JCM 5805. We also showed that LC-Plasma activates murine NK cells via dendritic cells activation^11^. Additionally, the oral administration of LC-Plasma strongly decreased pathogenesis in a murine parainfluenza virus infection model^12^. In some clinical studies, the intake of yogurt or supplements containing LC-Plasma has been reported to affect the activity of human pDCs and mitigate the symptoms of flu and common cold without adverse effects^13–15^. Hence, the activation of pDCs by LAB seems to be beneficial to defend against viral infection.

Recent research has suggested that in addition to their preventive effect against viral infection, pDCs also possess tumoricidal activities that are exerted directly in a granzyme B and TRAIL-dependent manner and indirectly through the activation of NK cells by type I IFN^16^. Since our previous report suggested that pDCs stimulated by LC-Plasma activate NK cells^11^, it is anticipated that LAB strains that activate pDCs have various immunostimulatory actions besides enhancing anti-viral activity.

Emulsifiers have recently been highlighted as candidate adjuvants for some viral vaccines and cancer immunotherapies. A recent report showed that the conjugation of the immunomodulatory molecule to an emulsifier, namely poly(ethylene glycol)-polylactide, effectively potentiated the induction of a Th1-dominant antigen-specific immune response as well as showing an antitumor effect^17^. Other reports have shown that synthetic phosphatidylcholine emulsifiers in combination with an inactivated influenza vaccine or a recombinant malaria antigen yielded enhanced immune responses as compared to the same antigens without adjuvant^18,19^. However, the emulsifiers used in these studies are artificially synthesised and have not been confirmed as safe for human use. Furthermore, although some cytokines related to the immune response were measured in these studies, the effect of emulsifiers on pDCs was not measured.

Therefore, in this study, we searched for safe emulsifiers that may enhance the potency of LAB against pDCs activation for the purpose of endowing LAB with a broad variety of functions. In addition to performing this search, we also explored the mechanism by which the emulsifiers enhance the potency of LAB.

## Results

### Specific sucrose fatty acid esters enhance the potency of LAB to activate pDCs

**Figure 1:**
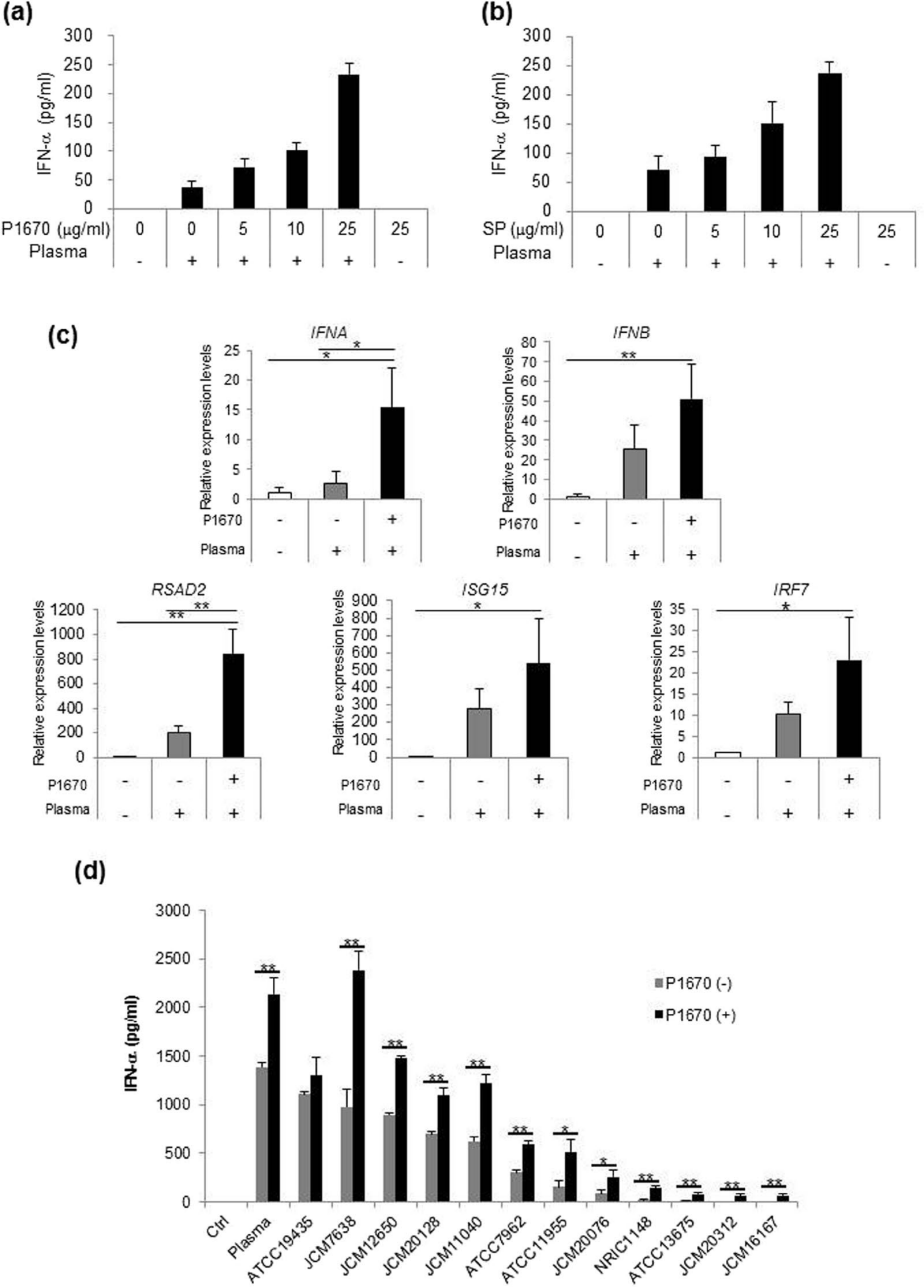
An emulsifier (P-1670) and its component in combination with the lactic acid bacteria induced IFN-α production and anti-viral gene expression. (**a**,**b**) LC-Plasma (2 mg/mL) was mixed with an equal volume of 0–50 mg/mL P-1670 or 0–50 mg/mL SP. Bone marrow-derived dendritic cells (BM-DCs) were treated with the above samples at a dilution of 1:100 in the culture medium and cultured for 24 h. The concentration of IFN-α in the culture medium was measured by enzyme-linked immunosorbent assay (ELISA). Assays were performed in triplicate wells. The data show the mean ± standard deviation (SD) for triplicate wells. (**c**) BM-DCs were cultured with 10 μg/mL LC-Plasma or 10 μg/mL LC-Plasma mixed with P-1670 for 16 h. Cells were harvested and their gene expression levels were measured by reverse transcription polymerase chain reaction. The data show the mean ± standard deviation (SD) for triplicate wells. The statistical significance of differences among treatment groups was measured using one-way analysis of variance (ANOVA) with Tukey’s post-hoc test (*p < 0.05, **p < 0.01). (**d**) BM-DCs were cultured with 10 μg/mL of a series of LAB strains or LAB strains mixed with P-1670 for 24 h. The concentration of IFN-α in the culture medium was measured by enzyme-linked immunosorbent assay (ELISA). Assays were performed in triplicate wells. The data show the mean ± SD for triplicate wells. The statistical significance between treatments with LAB strains with or without P-1670 was measured using Student’s *t*-test (*p < 0.05, **p < 0.01).

Emulsifiers are amphiphiles that consist of hydrophilic and hydrophobic groups. Several types of emulsifiers have hydroxyl groups modified with organic acids. During our search for emulsifiers that enhance the effects of LC-Plasma, we used emulsifiers with different hydrophobic and hydrophilic groups modified with or without organic acids as listed in Supplementary Table 1. As an index of pDC activity, IFN-α production by bone marrow-derived DCs (BM-DCs) treated with LC-Plasma mixed with the emulsifiers was measured. At first, for the purpose of estimating the effects of various emulsifiers, we used a variety of emulsifiers composed of triglyceride, polyglyceride and sucrose as hydrophilic groups and laurate, myristate, palmitate and stearate as hydrophobic groups. LC-Plasma in combination with RYOTO^®^ sugar ester P-1670 (P-1670) or RYOTO^®^ sugar ester S-1670 (S-1670) induced significantly higher IFN-α production compared to LC-Plasma alone (Table 1). Some of the other emulsifiers also induced higher IFN-α production in combination with LC-Plasma compared to LC-Plasma alone, but these differences were not significant. P-1670 and S-1670 are composed of sucrose as the hydrophilic group together with palmitate and stearate as the hydrophobic groups, respectively. We next measured the effect of an emulsifier composed of an unsaturated fatty acid, oleate, as the hydrophobic group and this emulsifier showed no effect (Supplementary Table 2). Although the emulsifiers that were composed of monoglyceride with or without an organic acid modification as the hydrophilic group induced significantly higher IFN-α production compared to LC-Plasma alone (Supplementary Table 3), the effect was smaller than that observed for sucrose. The most effective emulsifier was P-1670. Therefore, we next compared the dose dependency of P-1670, which showed the strongest activity and sucrose palmitate (SP) as the main component of P-1670, on IFN-α production. The results showed that both P-1670 and SP in combination with LC-Plasma induced IFN-α production in a dose-dependent manner at comparable concentrations (Fig. 1a,b). Therefore, the effect of P-1670 was considered to result from the activity of SP. We next investigated whether P-1670 could enhance the potency of LC-Plasma to induce gene expression related to anti-viral immunity. The gene expression levels of BM-DCs that were treated with LC-Plasma mixed with or without P-1670 for 16 h were measured. The expression levels of *Ifna* and *Rsad2* in cells treated with LC-Plasma mixed with P-1670 were significantly higher than those of cells treated with LC-Plasma alone and untreated cells. *Ifnb*, *Isg15* and *Irf7* were also significantly upregulated in cells that were treated with LC-Plasma mixed with P-1670 as compared to untreated cells, although only a modest upregulation of the expression of these three genes was observed in cells that were treated with LC-Plasma alone (Fig. 1c). These data showed that P1670 enhanced the potency of LC-Plasma to induce gene expression related to anti-viral immunity. In addition to LC-Plasma, some LAB strains can induce IFN-α production by BM-DCs. We next measured the action of P-1670 on some bacterial strains including *L. lactis* subsp. *lactis*, *L. lactis* subsp. *hordiniae*, *L. lactis* subsp. *cremoris* and *Enterococcus malodoratus* besides LC-Plasma. Interestingly, among fifteen LAB strains tested, twelve strains showed a significant induction of IFN-α production when mixed with P-1670 compared to the same LAB strains alone (Fig. 1d). These data suggested that P-1670 enhances the activity of multiple species of LAB to stimulate pDCs.

**Table 1:** IFN-α production by BM-DCs stimulated with LC-Plasma mixed with emulsifiers.

No.	Plasma	Emulsifier	IFN-α (pg/mL)	
1	−	−	1.8 ± 3.2	
2	+	−	60.4 ± 6.7	
3	+	P-1670	267.1 ± 58.5	**
4	+	S-1670	220.2 ± 30.8	**
5	+	M-1695	118.0 ± 14.6	
6	+	TRP-97RF	11.0 ± 13.9	
7	+	S-28D	117.5 ± 6.3	
8	+	M-7D	89.2 ± 24.8	
9	+	L-100	75.7 ± 8.7	

BM-DCs were cultured with 10 μg/mL of LC-Plasma or LC-Plasma mixed with emulsifiers for 24 h. The concentration of IFN-α in the culture medium was measured by ELISA. The data show the mean ± SD for triplicate wells. The statistical significance between different treatment groups was measured using one-way ANOVA with Dunnett’s post-hoc test for comparison to the LC-Plasma alone group (**p < 0.01).

### P-1670 enhances the downstream signaling effects induced by LC-Plasma to stimulate IFN-α production

**Figure 2:**
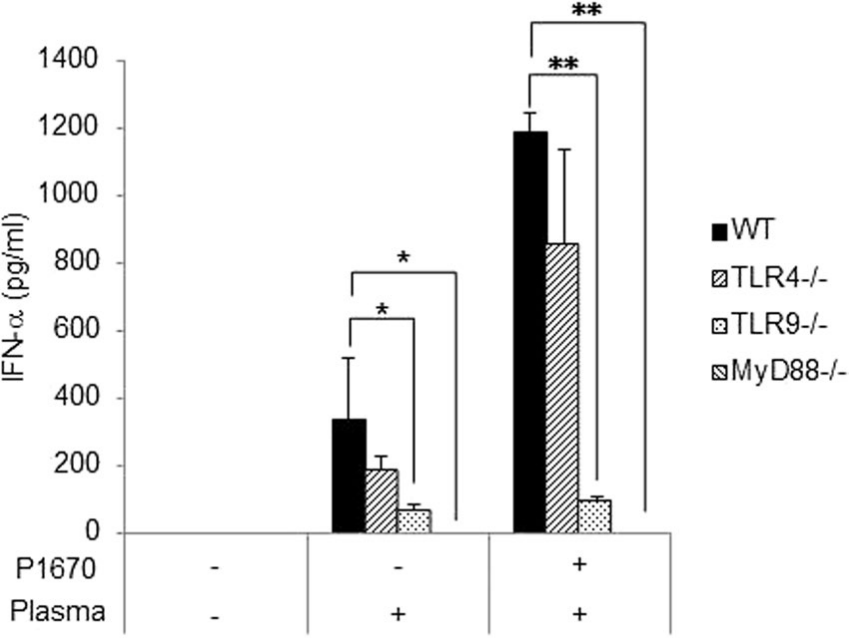
P-1670 enhances the signals of LC-Plasma for IFN-α production. BM-DCs were prepared from C57BL/6 J wild-type (WT), *Tlr4*^−/−^, *Tlr9*^−/−^ and *Myd88*^−/−^ mice. Cells were cultured with 10 μg/mL LC-Plasma or 10 μg/mL LC-Plasma mixed with P-1670 for 24 h. The concentration of IFN-α in the culture medium was measured by ELISA. The data show the mean ± SD for triplicate wells. The statistical significance of differences among the groups of mice was measured using one-way ANOVA with Tukey’s post-hoc test (*p < 0.05, **p < 0.01).

It has been reported that the induction of IFN-α production by LC-Plasma is completely impaired in *Tlr9*^−/−^ mice and partially impaired in *Tlr4*^−/−^ mice^10^. To investigate whether P-1670 compensates for defects related to the TLR signaling pathways, we investigated the effect of P1670 on IFN-α production elicited by LC-Plasma using BM-DCs from wild-type, *Tlr4*^−/−^, *Tlr9*^−/−^ and *Myd88*^−/−^ mice. As we reported previously, the induction of IFN-α production by LC-Plasma was decreased to some extent in *Tlr4*^−/−^ BM-DCs and it was almost abolished in *Tlr9*^−/−^ and *Myd88*^−/−^ BM-DCs that were treated with LC-Plasma alone. The same tendencies were observed when the same cell types were treated with LC-Plasma mixed with P-1670 (Fig. 2). These data suggested that the enhancing effect of P-1670 on pDCs stimulation does not compensate for defects in the TLR signaling pathway.

### The effect of P-1670 on cell surface activation markers of pDCs and mDCs

**Figure 3:**
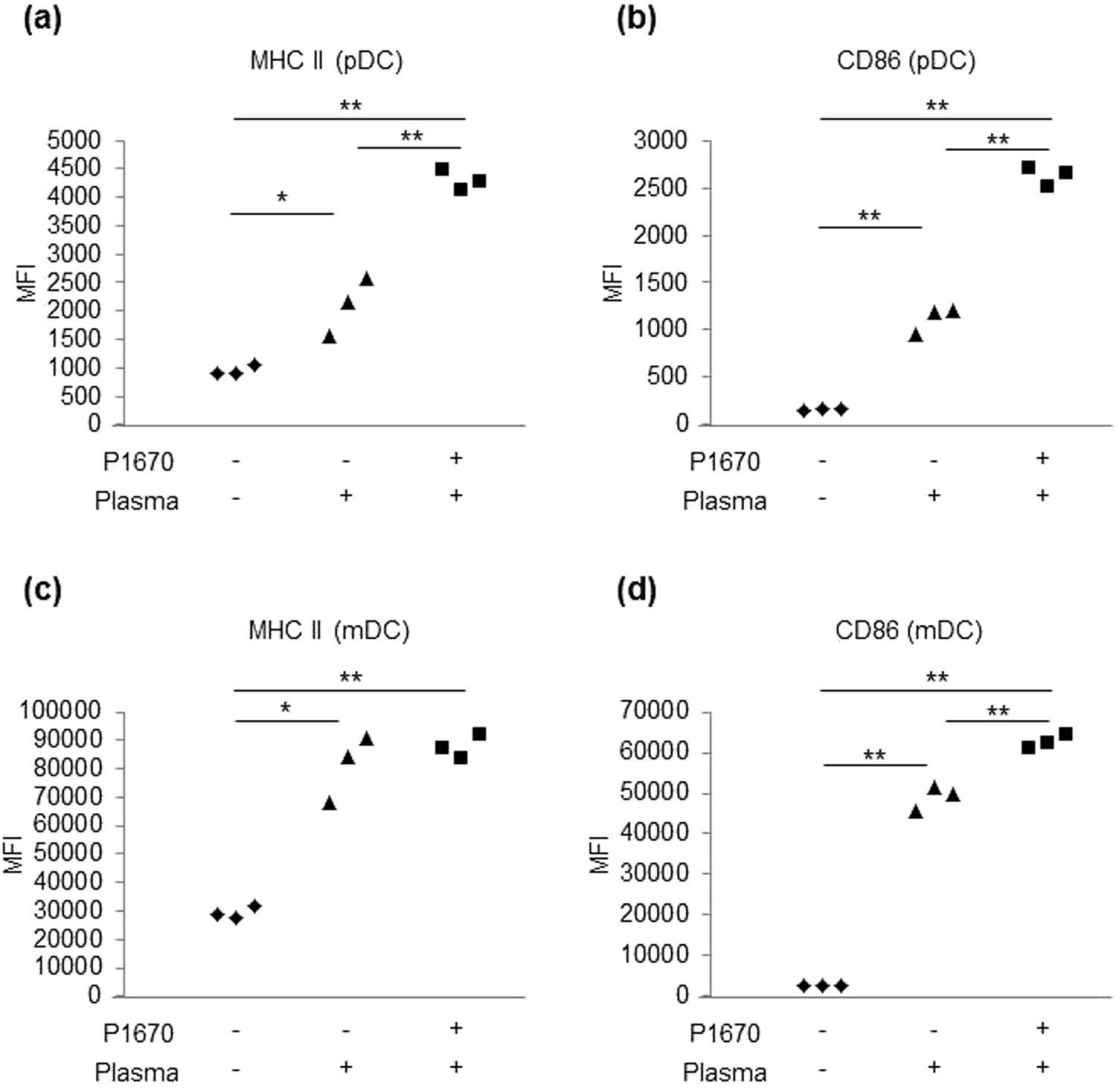
P-1670 mainly modulates the effect of LC-Plasma on the activity of plasmacytoid dendritic cells (pDCs). BM-DCs were cultured with 10 μg/mL of LC-Plasma or 10 μg/mL of LC-Plasma mixed with P-1670 for 24 h. Cells were evaluated for their expression level of cell surface markers by flow cytometry. (**a**,**b**) Cell surface markers on pDCs. (**c**,**d**) Cell surface markers on myeloid dendritic cells (mDCs). The numbers presented in the graph indicate median fluorescent intensity. Assays were performed in triplicate wells. The statistical significance of differences among treatment groups was measured using one-way ANOVA with Tukey’s post-hoc test (*p < 0.05, **p < 0.01).

BM-DCs induced by Flt-3L mainly contain pDCs and mDCs and our previous observations revealed that the activation of pDCs by LC-Plasma was significantly enhanced by the presence of mDCs in the cell cultures^10^. We next evaluated whether the emulsifier P-1670 could affect the activity of LC-Plasma towards pDCs or mDCs. BM-DCs were cultured with LC-Plasma alone or in combination with P-1670 for 24 h and then the cell surface expression of CD86 and MHC class II on pDCs and mDCs was determined by flow cytometry. In the pDC population, both MHC class II and CD86 were significantly upregulated by treatment with LC-Plasma mixed with P-1670 as compared to LC-Plasma alone (Fig. 3a,b). In the mDCs population, only CD86 was significantly upregulated by LC-Plasma mixed with P-1670 as compared to LC-Plasma alone (Fig. 3c,d) and the effect was smaller than that observed in the pDC population. These data suggested that P-1670 primarily modulates the effect of LC-Plasma on pDCs.

### The uptake of LC-Plasma by pDCs positively correlated with IFN-α production by BM-DCs

**Figure 4:**
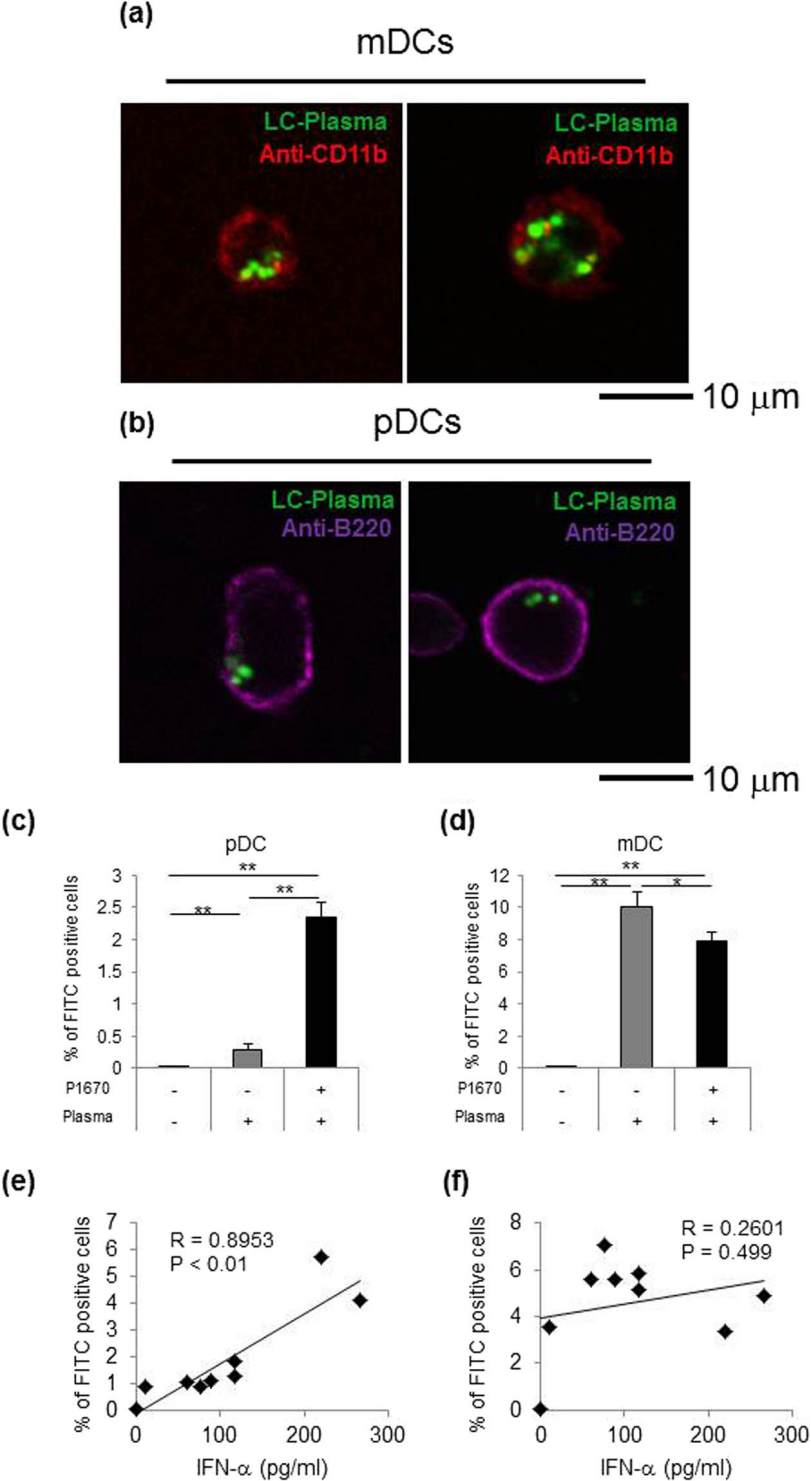
LC-Plasma uptake by pDCs positively correlated with IFN-α production by BM-DCs. (**a,b**) Representative confocal fluorescence images of pHrodo™ Green (Green)-labelled LC-Plasma internalised into mDCs (left) and pDCs (right). The surfaces of mDCs and pDCs were stained with APC-Cy7-labelled anti-CD11b and APC-labelled anti-B220 antibodies, respectively. Bar, 10 μm. (**c,d**) pDCs and mDCs purified by fluorescence-activated cell sorting were cultured with 10 μg/mL of LC-Plasma conjugated with pHrodo™ Green or 10 μg/mL of LC-Plasma mixed with P-1670 for 18 h. Cells were harvested and the percentage of cells that emitted fluorescence was determined by flow cytometry analysis. The data show the mean ± SD for triplicate wells. The statistical significance of differences among treatment groups was measured using one-way ANOVA with Tukey’s post-hoc test (*p < 0.05, **p < 0.01). (**e** and **f**) Correlations between IFN-α production as described in Table 1 and (**e**) the percentage of pDCs that took up lactic acid bacteria as described in Table 2 or (**f**) the percentage of mDCs that took up lactic acid bacteria as described in Table 3 were measured. *p* < 0.05, the inset corresponds to Pearson’s correlation coefficient (*r*) and the corresponding *p* value.

In the previous report, the uptake of LC-Plasma by pDCs was suggested to be essential for the LC-Plasma mediated stimulation of pDCs^10^. Therefore, it is assumed that P-1670 may enhance the uptake of LC-Plasma into pDCs. Fluorescent dye-labelled LC-Plasma mixed with/without P-1670 was added to pDCs and mDCs, respectively, which were purified using a cell sorter. Qualitative observations of whether pDCs and mDCs captured LC-Plasma were made by confocal laser scanning microscopy. Furthermore, the percentage amount of pDCs and mDCs that took up LC-Plasma was quantitatively determined by flow cytometry analysis. The confocal fluorescence images confirmed that LC-Plasma was internalised into pDCs and mDCs (Fig. 4a,b). The percentage of pDCs that took up LC-Plasma was drastically increased by the addition of LC-Plasma mixed with P-1670 as compared to LC-Plasma alone (Fig. 4c). However, the ratio of mDCs that took up LC-Plasma was not affected by the addition of P-1670 (Fig. 4d). These data suggest that P-1670 selectively affects the uptake of LC-Plasma by pDCs, but not by mDCs. To determine whether the enhancement of LC-Plasma uptake by pDC is limited to P-1670 or represents a general attribute of various emulsifiers, the other emulsifiers listed in Table 1 were also tested. The emulsifier S-1670, which is mainly composed of sucrose fatty acid esters, was found to be effective as well as P-1670 (Table 2). The other emulsifiers could not enhance uptake by pDCs. In the case of mDCs, only L-100 enhanced LC-Plasma uptake, while TRP-97RF and S-1670 attenuated LC-Plasma uptake (Table 3). We next performed correlational analyses between LC-Plasma uptake and IFN-α production using the data shown in Tables 1–3. Interestingly, the uptake of LC-Plasma by pDCs positively and significantly correlated with IFN-α production by BM-DCs, while the uptake of LC-Plasma by mDCs was not significantly correlated with IFN-α production by BM-DCs (Fig. 4e,f). These data suggested that emulsifiers enhance IFN-α production by BM-DCs via the enhancement of LC-Plasma uptake by pDCs.

**Table 2:** LC-Plasma uptake by pDCs stimulated with LC-Plasma mixed with emulsifiers.

No.	Plasma	Emulsifier	% of FITC positive cells	
1	−	−	0.0 ± 0.0	
2	+	−	1.0 ± 0.1	
3	+	P-1670	4.1 ± 0.1	**
4	+	S-1670	5.8 ± 0.4	**
5	+	M-1695	1.8 ± 0.2	
6	+	TRP-97RF	0.9 ± 0.6	
7	+	S-28D	1.3 ± 0.0	
8	+	M-7D	1.1 ± 0.2	
9	+	L-100	0.9 ± 0.4	

pDCs were cultured with 10 μg/mL of pHrodo™-conjugated LC-Plasma or 10 μg/mL of pHrodo™-conjugated LC-Plasma mixed with emulsifiers for 18 h. Cells were harvested and the percentage of cells that emitted fluorescence was determined by flow cytometry analysis. The data show the mean ± SD for triplicate wells. The statistical significance of differences among treatment groups was measured using one-way ANOVA with Dunnett’s post-hoc test for comparison to the LC-Plasma alone group (**p < 0.01).

**Table 3:** LC-Plasma uptake by mDCs stimulated with LC-Plasma mixed with emulsifiers.

No.	Plasma	Emulsifier	% of FITC-positive cells	
1	−	−	0.0 ± 0.0	
2	+	−	5.6 ± 0.1	
3	+	P-1670	4.9 ± 0.2	
4	+	S-1670	3.4 ± 0.1	**
5	+	M-1695	5.1 ± 1.2	
6	+	TRP-97RF	3.6 ± 0.3	**
7	+	S-28D	5.9 ± 0.0	
8	+	M-7D	5.6 ± 0.2	
9	+	L-100	7.1 ± 0.2	*

mDCs were cultured with 10 μg/mL of pHrodo™-conjugated LC-Plasma or 10 μg/mL of pHrodo™-conjugated LC-Plasma mixed with emulsifiers for 18 h. The cells were harvested and the percentage of cells that emitted fluorescence was determined by flow cytometry analysis. The data show the mean ± SD for triplicate wells. The statistical significance of differences among treatment groups was measured using one-way ANOVA with Dunnett’s post-hoc test for comparison to the LC-Plasma alone group (*p < 0.05, **p < 0.01).

### Effects of P-1670 on pDCs activity and anti-viral immunity *in vivo*

**Figure 5:**
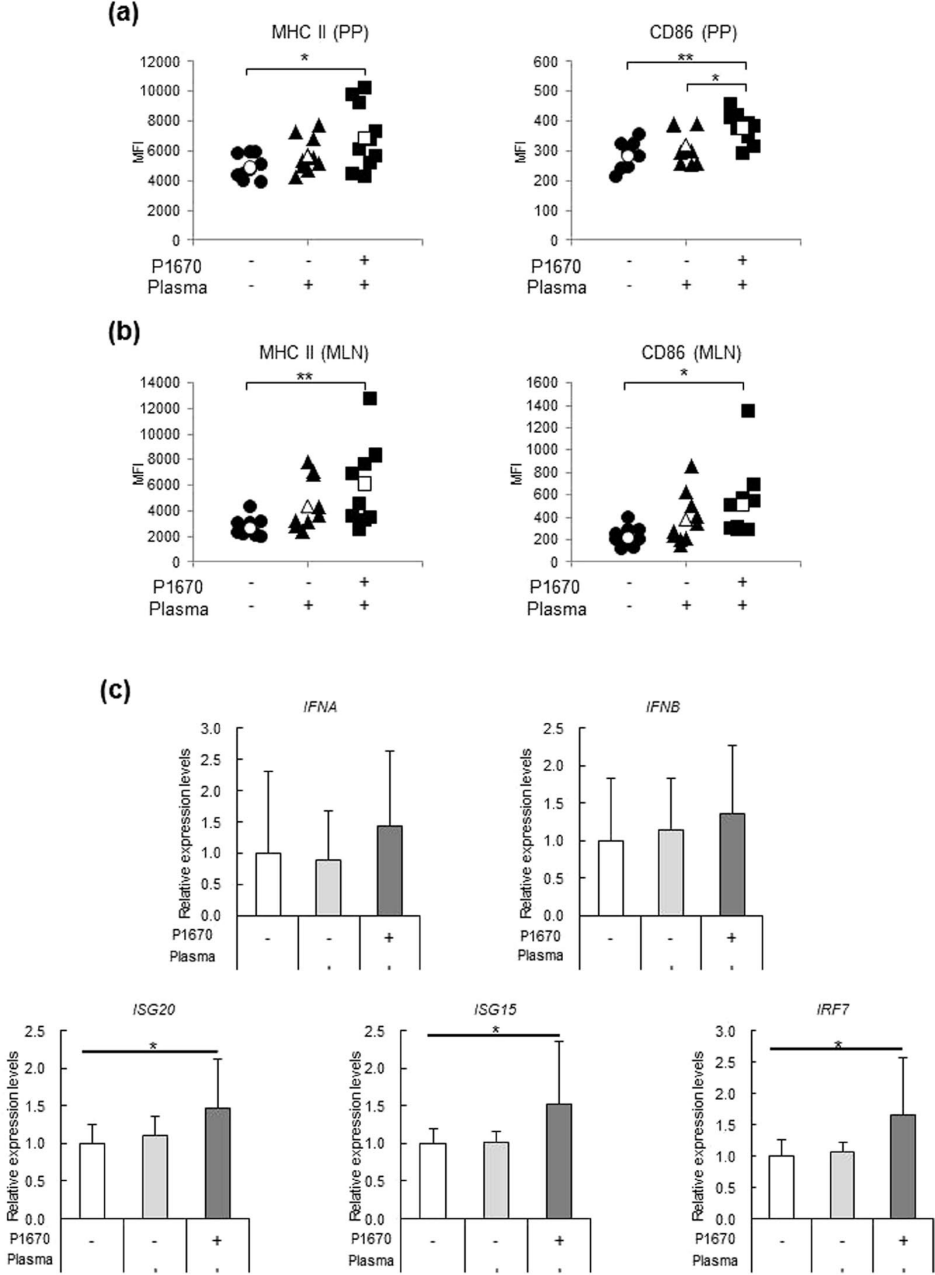
Effects of P-1670 on *pDCs activity and* anti-viral immunity *in vivo*. (**a,b**) Mice were randomly divided into three groups consisting of ten mice each; control, LC-Plasma and LC-Plasma mixed with P-1670. Each group was orally administered with the following treatments: distilled water for the control group, 1 mg of LC-Plasma for the LC-Plasma group and 1 mg of LC-Plasma mixed with P1670 for the LC-Plasma mixed with P-1670 group. Twenty-four hours after the single administration, the mice were sacrificed and the cell surface expression of CD86 and MHC class II on the pDCs of Peyer’s patches and mesenteric lymph nodes was measured by flow cytometry. The data show the mean ± SD. The white-coloured marks show the average of each group. The statistical significance of differences among treatment groups was measured using one-way ANOVA with Tukey’s post-hoc test (*p < 0.05, **p < 0.01). (**c**) Mice were randomly divided into three groups consisting of ten mice each; control, LC-Plasma and LC-Plasma mixed with P-1670. Each group was fed as follows: AIN93G for the control group, AIN93G blended with 0.033 mg/g dry weight of LC-Plasma for the LC-Plasma group and AIN93G blended with 0.117 mg/g dry weight of LC-Plasma mixed with P-1670 for the LC-Plasma mixed with P-1670 group. Two weeks later, the mice were sacrificed and their lungs were excised. The gene expression levels of *Ifna*, *Ifnb*, *Isg20*, *Isg15* and *Irf7* were measured by polymerase chain reaction. The data show the mean ± SD. The statistical significance of differences among treatment groups was measured using one-way ANOVA with Tukey’s post-hoc test (*p < 0.05).

We previously reported that the oral administration of LC-Plasma to mice activated intestinal pDCs^10^ and strongly inhibited the development of symptoms of parainfluenza virus infection via modulating anti-viral immunity in the lung^12^. To investigate whether P-1670 could also enhance anti-viral immunity *in vivo*, LC-Plasma mixed with P-1670 was orally administered to mice, the activation of intestinal pDCs as well as the expression of genes related to anti-viral immunity in lung tissue were measured. For the measurement of intestinal pDCs activation, mice were randomly divided into three groups; control, LC-Plasma and LC-Plasma mixed with P-1670. Twenty-four hours after the single administration, the mice were sacrificed and the cell surface expression of CD86 and MHC class II on pDCs was measured. In the Peyer’s patches (PPs), both MHC class II and CD86 were slightly upregulated by the LC-Plasma treatment as compared to the control group, but the difference was not statistically significant in this experiment. However, a significant upregulation of these molecules was detected in the group treated with LC-Plasma mixed with P-1670 as compared to the control (Fig. 5a). In mesenteric lymph nodes (MLN), similarly to PPs, both MHC class II and CD86 were significantly upregulated by the treatment of LC-Plasma mixed with P-1670 as compared to the control (Fig. 5b). These data suggested that P-1670 could enhance the activation of intestinal pDCs by LC-Plasma. For the measurement of anti-viral genes in lung tissue, mice were randomly divided into three groups; control, LC-Plasma and LC-Plasma mixed with P-1670. The dose of LC-Plasma administered in this study was considered to have a weak effect in our previous study. After 2 weeks, the mice were sacrificed and their gene expression in lung was measured. The expression levels of *Ifna* and *Ifnb* were low and no significant difference between the groups was detected, whereas the expression levels of *Isg15*, *Isg20* and *Irf7* were significantly higher only in the group treated with LC-Plasma mixed with P-1670 as compared to the control group (Fig. 5c). These data suggested that P-1670 could enhance the anti-viral immunity stimulating effect of LC-Plasma *in vivo*.

## Discussion

When they are activated, pDCs secrete large amounts of type I interferons, which are crucial to establish the anti-viral state and lead to further innate and adaptive immune responses^20^. We previously showed that LC-Plasma exerts anti-viral effects via the activation of pDCs and their subsequent IFN-α production. Herein, we showed that emulsifiers enhanced the potency of LC-Plasma to activate pDCs and induce IFN-α production. Interestingly, the enhancement of pDCs activity by LAB mixed with emulsifiers was induced not only by the LAB strains that could activate pDCs when they were administered without emulsifiers but also by the LAB strains that lacked that ability (Fig. 1d). pDCs are primarily activated through the engagement of endosomally located TLR-7 and TLR-9 by ssRNA^21,22^ or by nonmethylated and CpG DNA^23,24^, respectively. We previously reported that DNA extracted from LC-Plasma could activate pDCs. Therefore, for activating pDCs, it is considered that LAB need to be taken up into pDCs and to have specific structures of RNA or DNA for binding TLRs. Herein, we showed that the ratio of pDCs that took up LC-Plasma correlated with the amount of IFN-α production by BM-DCs. Notably, the promotion of LC-Plasma uptake by emulsifiers was observed only in pDCs and not in mDCs. Therefore, it is considered that the emulsifiers induced synergistic pDCs activation via the promotion of LAB uptake by pDCs. However, it remains unrevealed why emulsifiers enhance LAB uptake by pDCs. It is assumed that the adhesion intensity of LAB to pDCs might be enhanced and the recognition of LAB by pDCs was improved by the action of the emulsifier. Since several emulsifiers promoted pDCs activation by LAB with differing intensities, the structures of the emulsifiers seemed to affect the adhesion intensity and recognition of LAB. The data reported herein suggested that, for an emulsifier to promote pDCs activation by LAB, the hydrophobic group should preferably be a saturated fatty acid with a carbon chain length of C16 or longer and the hydrophilic group should preferably be sucrose.

Some studies have evaluated whether synergistic immune activation could occur with the simultaneous activation of TLRs^25–28^. For example, the simultaneous treatment of mice with ligands for TLR2/6 and TLR9 conferred synergistic protection against microbial disease in the lung epithelium^25^. Moreover, co-stimulation with TLR9 and TLR3 ligands synergistically activated genes that are primarily associated with immune function^26^. In this study, the data obtained using BM-DCs derived from TLR4 and TLR9 knockout mice suggested that emulsifiers enhanced the activation of the TLR9 signalling pathway by LC-Plasma. However, it remains unclear whether emulsifiers affect the other TLRs, since TLR9 is localized intracellularly and LC-Plasma activates TLR9 signaling, P1670 seems to promote the uptake of LC-Plasma by pDCs and enhance the accessibility of the DNA of LC-Plasma to TLR9. TLR ligands have also shown potential as immunotherapeutics for the treatment of cancer, allergy, and infectious diseases^29–32^. Especially, CpG oligodeoxynucleotides as TLR9 ligands are likely to be useful as an adjuvant for cancer treatment, in which their mode of action depends on cross-talk between dendritic cell subsets^30^. However, as for the safety of CpG oligodeoxynucleotides, clinical trials have indicated that they could induce the frequency and severity of local adverse events and systemic symptoms^29^. Both the LAB strain LC-Plasma and the emulsifiers employed in this study have been confirmed as safe.

In the present study, we also demonstrated the synergistic activation of intestinal pDCs and the induction of the expression of anti-viral genes in lung by the oral administration of LC-Plasma in combination with emulsifiers *in vivo*. We observed a statistically significant increase in the expression of *Isg15*, *Isg20* and *Irf7* in lung, although there was no significant change in the expression of *Ifna* and *Ifnb*. This localised increase in the expression of anti-viral genes shows that LC-Plasma has an effect on the lung despite its distance from the small intestine, which LC-Plasma firstly contacts. It has been reported that the host intestinal microbiota can induce IFN-β production from peritoneal and alveolar macrophages and thereby elevate the expression of ISGs in lung following respiratory influenza virus infection^33^. It has also been reported that the host commensal microbiota is a critical regulator of the generation of virus-specific CD4^+^ and CD8^+^ T cells as well as the antibody response in lung tissue following respiratory influenza virus infection and these regulatory effects of the host microbiota vary depending on its composition^34^. These reports and our observations in the present study might imply that there is a close relationship between the bronchus-associated and gut-associated lymphoid tissues. Although IFN proteins were not detected in plasma in this study because their concentration was very low, given that intestinal pDCs were activated by LC-Plasma, it seems possible that IFNs produced in the intestine might affect the expression of ISGs in lung via the blood circulatory system. ISGs are involved in the inhibition of viral replication and release^3^. ISG15 not only inhibits the replication and release of viruses from infected cells but also functions as an extracellular signaling molecule that stimulates IFN-γ secretion^35,36^. ISG20 acts as an innate anti-hepatitis B virus and anti-influenza A virus effector that selectively blocks the replication of those viruses^37,38^. IRF7, which is an ISG, not only functions as a modulator of IFN-α and IFN-β production but might regulate the development of granulocyte-like myeloid derived suppressor cells in cancer^39,40^. In addition to their anti-viral activity, pDCs are also involved in anti-cancer immunity. It has been reported that vaccination using CpG-B-activated tumor antigen-presenting pDCs promotes the intratumoral recruitment of immune cells including antitumor cytotoxic T lymphocytes^41^. Another report showed that pDCs can elicit cytotoxic immune responses towards tumor cells via the activation of cytotoxic T lymphocytes and innate immune cells^42^. pDCs-dependent antigen-driven T cell proliferation was reported to be suppressed by the attenuation of IFN-α production^43^. The use of LC-Plasma in combination with emulsifiers is anticipated to be useful as an adjuvant for certain anti-viral and anti-cancer therapies. In this study, no significant activation of intestinal mDCs by the oral administration of LC-Plasma in combination with emulsifiers was detected as compared to LC-Plasma alone (Supplementary Figure 1). These data and the *in vitro* data suggest that emulsifiers do not activate mDCs.

Although the dose of emulsifiers used was larger than the dose normally contained in a product for daily use, the data showed that emulsifiers could enhance the immunomodulative effect of LC-Plasma *in vivo*. Future studies will be needed to confirm whether some LAB strains in combination with certain emulsifiers could be used as adjuvants to enhance the effects of some viral and cancer immunotherapies.

## Methods

### Animals

All animal care and experimental procedures were performed in accordance with the guidelines of the Committee for Animal Experimentation at Kirin Company. These studies were approved by the Committee for Animal Experimentation at Kirin Company. Mice were kept in a room maintained on a 12:12-h light-dark cycle at a temperature of 23 ± 1 °C. Mice for BM-DCs assays were provided with a standard mouse chow (CE-2; CLEA Japan, Tokyo, Japan) and mice for the *in vivo* study were provided with AIN-93G (Oriental Yeast, Tokyo, Japan). BALB/c, C57BL/6 J and C57 BL/6 J *Tlr4*^−/−^, *Tlr9*^−/−^ and *Myd88*^−/−^ mice were obtained from Charles River Laboratories Japan (Kanagawa, Japan). 129/Sv mice were obtained from Japan SLC, Inc. (Shizuoka, Japan).

### BM-DC culture and assay

Flt-3L-induced BM-DCs were generated as previously described^44^. In brief, bone marrow cells were extracted from mice and erythrocytes were removed by a brief exposure to red blood cell lysis buffer (Sigma-Aldrich Japan, Tokyo, Japan). Cells were cultured at a density of 5 × 10^5^ cells/mL for 7 days in Roswell Park Memorial Institute 1640 medium supplemented with 1 mM sodium pyruvate (Invitrogen, Carlsbad, CA, USA), 2.5 mM HEPES (Invitrogen), penicillin-streptomycin (Invitrogen), 50 mM β-mercaptoethanol (Invitrogen), 10% foetal calf serum and 100 ng/mL Flt-3L (R&D systems, Minneapolis, MN, USA). LAB with or without emulsifiers were added at the concentrations indicated in the figure legends and cultured for 24 h.

### LAB strains and emulsifiers

The LAB strains tested in this study were purchased from the collections held at the Japan Collection of Microorganisms (JCM), American Type Culture Collection (ATCC), and Culture Collection Center of Tokyo University of Agriculture (NRIC). Cultures of LAB strains were grown at 30 °C or 37 °C for 48 h in MRS broth (Oxoid, Hampshire, UK), GAM broth (Nissui, Tokyo, Japan), or M17 broth (Oxoid, Hampshire, UK) according to each manufacturer’s instructions. Cultured LAB strains were washed twice with sterile distilled water, heat-killed at 100 °C, lyophilised, and suspended in phosphate-buffered saline. The emulsifiers tested in this study were obtained from two companies. A series of RYOTO^®^ esters and Emulsie P-100 emulsifiers were obtained from Mitsubishi-Kagaku Foods Corporation (Tokyo, Japan) and a series of Poem emulsifiers were obtained from RIKEN VITAMIN (Tokyo, Japan). Sucrose palmitate was obtained from Sigma-Aldrich Japan. LAB strains mixed with emulsifiers were prepared as follows, unless otherwise described in the figure legends. Emulsifiers were dissolved in distilled water at 5 mg/mL with heating at 60 °C and equal volumes of LAB at 2 mg/mL were added and incubated for 30 min.

### RNA isolation and real-time quantitative reverse transcription polymerase chain reaction (RT-PCR)

Gene expression levels in BM-DCs and lung tissue were measured by real-time quantitative RT-PCR. Total RNA was extracted using TRIzol^®^ Reagent (Ambion, Waltham, MA, USA). After the extraction of total RNA, a purification step was performed using an RNeasy^®^ micro kit (Qiagen, Hilden, Germany) for BM-DCs and an RNeasy^®^ mini kit for lung (Qiagen). To synthesise cDNA, purified total RNA samples were reverse transcribed using the SuperScript^®^ VILO™ cDNA Synthesis Kit (Invitrogen, Carlsbad, CA, USA). The quantification of relative RNA levels was performed with SYBR^®^ Green real-time PCR technology (Applied Biosystems, Foster City, CA, USA). Gene expression data for BM-DCs and lung were normalised to the expression of 18 S rRNA and ribosomal protein, large, P0 (P0) RNA, respectively. Specific forward and reverse primer pairs were as follows.

*P0* F: GCGTCCTGGCATTGTCTGTG

*P0* R: TCCTCATCTGATTCCTCCGACTC

*Ifna* F: AGCAGGTGGGGGTGCAGGAA

*Ifna* R: ACCACCTCCCAGGCACAGGG

*Ifnb* F: TCAGAATGAGTGGTGGTTGC

*Ifnb* R: GACCTTTCAAATGCAGTAGATTCA

*Isg20* F: GTCACGCCTCAGCACATGGT

*Isg20* R: CCACCAGCTTGCCTTTCAGAA

*Isg15* F: GAGCTAGAGCCTGCAGCAAT

*Isg15* R: TTCTGGGCAATCTGCTTCTT

*Irf7* F: ACAGGGCGTTTTATCTTGCG

*Irf7* R: TCCAAGCTCCCGGCTAAGT

*Rsad2* F: CTTCAACGTGGACGAAGACA

*Rsad2* R: GACGCTCCAAGAATGTTTCA

### Flow cytometry analysis

BM-DCs for flow cytometry were stained with fluorescent dye-conjugated antibodies (MHC class II-FITC, CD86-PE, CD11c-PE-Cy7, B220-APC and CD11b-APC-Cy7). After staining, the cells were washed twice with fluorescence-activated cell sorting (FACS) buffer (0.5% BSA in phosphate-buffered saline) and suspended in 4% paraformaldehyde for FACS analysis. Data were collected using a FACSCanto™ II flow cytometer (BD Biosciences, San Jose, CA, USA) and analysed by FCS Express™ software (De Novo Software, Glendale, CA, USA) to determine the abundance of MHC class II and CD86 on the surfaces of gated cells. BM-derived Flt-3L-induced pDCs and mDCs were defined as CD11c^+^ CD11b^−^ B220^+^ and CD11c^+^ CD11b^+^ B220, respectively. The following fluorescence-conjugated anti-mouse monoclonal antibodies were purchased from Thermo Fisher Scientific (Waltham, MA, USA): B220 (RA3-6B2), CD11c (N418), MHC class II (M5/114.15.2) and CD86 (GL1). CD11b (M1/70) was purchased from BD Biosciences (San Diego, CA, USA).

### Purification of pDCs and mDCs

pDCs and mDCs from BM-DCs culture were sorted using a FACSAria™ flow cytometer (BD Biosciences). BM-DCs were stained with fluorescent dye-conjugated antibodies as described above. After staining, the cells were washed twice with FACS buffer and sorted using a FACSAria™ flow cytometer. pDCs and mDCs were defined as CD11c^+^ CD11b^−^ B220^+^ and CD11c^+^ CD11b^+^ B220^−^, respectively. The purity of the sorted pDCs and mDCs was more than 95%.

### Measurement of the percentage of DCs that took up LAB

LAB strains conjugated with pHrodo™ Green STP ester (Thermo Fisher Scientific) were prepared from lyophilised LAB according to the manufacturer’s protocol. The purified pDCs and mDCs were cultured with 10 μg/mL of the pHrodo^TM^ Green-conjugated LAB for 20 h. The cells were washed twice with FACS buffer and suspended in FACS buffer for analysis. The percentage of cells that took up the LAB was determined by flow cytometry analysis and the cells were qualitatively observed by confocal laser scanning microscopy (FV1000, Olympus, Tokyo, Japan) using a UPlanSApo 40× lens (Olympus, N.A. 0.95). For confocal laser scanning fluorescence imaging of both pDCs and mDCs, the pHrodo™ Green dye was excited with a 473-nm laser and the fluorescent emission between 500 and 600 nm was monitored. For immunocytochemical imaging, anti-CD11b-APC-Cy7 antibody was excited at 635 nm and the fluorescent emission between 655 and 755 nm was monitored, while anti-B220-APC was excited at 635 nm and the fluorescent emission between 655 and 755 nm was monitored.

### *In vivo* study

For the measurement of intestinal pDCs activation, 6-week-old female C57BL/6 J mice were acclimatised for 1 week with free access to water and a basic diet, namely AIN93G chow (Oriental Yeast). The mice were divided into three groups consisting of ten mice each. The groups were orally administered with the following treatments: distilled water for the control group, 1 mg of LC-Plasma for the LC-Plasma group and 1 mg of LC-Plasma mixed with 0.5% P1670 for the LC-Plasma mixed with P-1670 group. Twenty-four hours after the single administration, the mice were sacrificed and their PPs and MLNs were excised. The cell surface expression of MHC class II and CD86 on the pDCs of each tissue was measured by flow cytometry. To measure the expression of anti-viral genes in lung tissue, 5-week-old female BALB/c mice were acclimatised for 1 week with free access to water and AIN93G chow. The mice were divided into three groups consisting of ten mice each. Each group was fed as follows: AIN93G for the control group, AIN93G blended with 0.033 mg/g dry weight of LC-Plasma for the LC-Plasma group and AIN93G blended with 0.117 mg/g dry weight of LC-Plasma mixed with P-1670 for the LC-Plasma mixed with P-1670 group. Two weeks later, the mice were sacrificed and their lungs were excised. The gene expression levels of *Ifna, Ifnb, Isg20, Isg15* and *Irf7* were measured by RT-PCR.

### Statistical analysis

Statistical comparisons were performed using one-way analysis of variance with Tukey’s test or Dunnett’s test for post-hoc comparisons, except for the results in Fig. 1(d), for which a standard Student’s *t* test was used. *p*-values of < 0.05 were considered to be statistically significant.

## Electronic supplementary material

Supplementary Tables and Figure
