# Supplementary material for: Enhancement of immunomodulative effect of lactic acid bacteria on plasmacytoid dendritic cells with sucrose palmitate
Source: Sci Rep. 2018 Feb 16;8:3147. doi: 10.1038/s41598-018-21527-2 (PMC5816640; doi:10.1038/s41598-018-21527-2)
Supplement: Supplementary file 1 — Supplementary Tables and Figure [file 41598_2018_21527_MOESM1_ESM.pdf]

Supplementary Information

Title
Enhancement of immunomodulative effect of lactic acid bacteria on plasmacytoid dendritic cells with sucrose palmitate

Authors
Masaya Kanayama[1,†,*] Yukiko Kato[1,†] Toshikazu Tsuji[2] Yuki Konoeda[2] Akiko Hashimoto[1] Osamu Kanauchi[1] Toshio Fujii[1] Daisuke Fujiwara[2]

† These authors equally contributed to this pater
* Corresponding author

Masaya Kanayama, Kirin Company, Limited, Research Laboratories for Health Science and Food Technologies, 1-13-5, Fukuura, Kanazawa-ku, Yokohama-city 236-0004, Japan; Tel.:+81-45-330-9004; Fax.:+81-45-788-4047;
e-mail: Masaya_Kanayama@Kirin.co.jp

Emulsifier	Hydrophilic group	Hydrophobic group	Organic acid
Emulsie P-100<br>(P-100)	monoglyceride	stearate and palmitate	-
Poem W-60<br>(W-60)	monoglyceride	stearate	tartrate
Poem B-15V<br>(B-15V)	monoglyceride	saturated fatty acid	succinate
Poem BS-20<br>(BS-20)	monoglyceride	saturated fatty acid	succinate
Poem TRP-97RF<br>(TRP-97RF)	triglyceride	palmitate	-
RYOTO sugar ester P-1670<br>(P-1670)	sucrose	palmitate	-
RYOTO sugar ester S-1670<br>(S-1670)	sucrose	stearate	-
RYOTO sugar ester M-1695<br>(M-1695)	sucrose	myristate	-
RYOTO sugar ester O-1570<br>(O-1570)	sucrose	oleate	-
RYOTO polyglycerol ester S-28D<br>(S-28D)	polyglyceride	stearate	-
RYOTO polyglycerol ester M-7D<br>(M-7D)	polyglyceride	myristate	-
RYOTO polyglycerol ester L-100<br>(L-100)	polyglyceride	laurate	-

Supplementary Table 1 Emulsifiers used in this study.
The major compounds contained in the tested emulsifiers are listed.

No.	Plasma	Emulsifier	IFN-α (pg/mL)			
1	-	-	0.0	±	0.0	
2	+	-	71.3	±	6.0	
3	+	P1670	219.7	±	49.5	**
4	+	S1670	151.5	±	17.9	**
5	+	O1570	49.8	±	4.8	

Supplementary Table 2 IFN-α production by BM-DCs stimulated with LC-Plasma mixed with emulsifiers. BM-DCs were cultured with 10 μg/mL of LC-Plasma or LC-Plasma mixed with emulsifiers for 24 h. The concentration of IFN-α in the culture medium was measured by ELISA. The data show the mean ± SD for triplicate wells. The statistical significance of differences among treatment groups was measured using one-way ANOVA with Dunnett's post-hoc test for comparison to the LC-Plasma alone group (**$p < 0.01$).

No.	Plasma	Emulsifier	IFN-α (pg/mL)			
1	-	-	0.0	±	0.0	
2	+	-	104.4	±	10.1	
3	+	P-100	128.5	±	11.7	
4	+	W-60	144.1	±	9.1	*
5	+	B-15V	156.3	±	21.5	**
6	+	BS-20	147.1	±	10.1	**

Supplementary Table 3 IFN-α production by BM-DCs stimulated with LC-Plasma mixed with emulsifiers. BM-DCs were cultured with 10 μg/mL of LC-Plasma or LC-Plasma mixed with emulsifiers for 24 h. The concentration of IFN-α in the culture medium was measured by ELISA. The data show the mean ± SD for triplicate wells. The statistical significance of differences among treatment groups was measured using one-way ANOVA with Dunnett's post-hoc test for comparison to the LC-Plasma alone group (*p < 0.05, **p < 0.01).

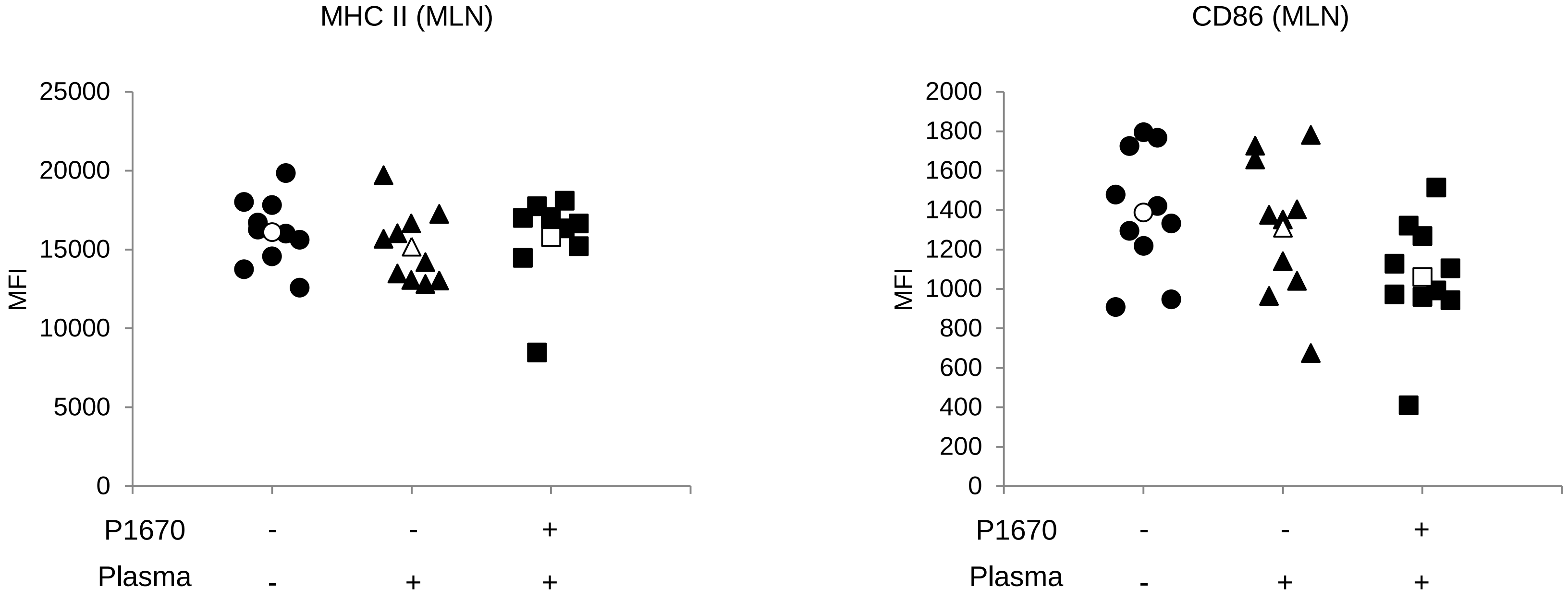

Supplementary Figure 1 Effects of P-1670 on mDCs activity in vivo. Mice were randomly divided into three groups consisting of ten mice each; control, LC-Plasma, and LC-Plasma mixed with P-1670. Each group was orally administered with the following treatments: distilled water for the control group, 1 mg of LC-Plasma for the LC-Plasma group, and 1 mg of LC-Plasma mixed with P1670 for the LC-Plasma mixed with P-1670 group. Twenty-four hours after the single administration, the mice were sacrificed and the cell surface expression of CD86 and MHC class II on the mDCs of mesenteric lymph nodes was measured by flow cytometry. The data show the mean ± SD. The white-coloured marks show the average of each group.
